# Supplementary material for: Understanding Vaccine Hesitancy in Vietnamese Fish Farmers
Source: Antibiotics (Basel). 2022 Jun 30;11(7):878. doi: 10.3390/antibiotics11070878 (PMC9312173; doi:10.3390/antibiotics11070878)
Supplement: Supplementary file 1 [file antibiotics-11-00878-s001.zip › antibiotics-1764635-supplementary.pdf]

Supplementary materials

Confirmatory principle components factor analysis was conducted for the three BMQ attitude measures (chemical treatment of water, antibiotics and vaccination) specifying two factors to be extracted and using Direct Oblimin rotation (as necessity and concerns are expected to be negatively correlated). Results are shown in Supplementary Table S1.

Supplementary Table S1: Confirmatory factor analysis of the BMQ, factor loadings and variance explained

	BMQ CT		BMQ AB		BMQ VAC	
	Nec	Conc	Nec	Conc	Nec	Conc
The health of my fish depends on the use CT/AB/VAC on my farm.	.776		.710		.673	
Having to use CT/AB/VAC on my fish farm worries me.		.528		.539		.563
Without CT/AB/VAC the fish on my farm would be very sick.	.672		.545		.625	
I sometimes worry about the long-term effects of CT/AB/VAC on my farm.		.672		.752		.741
CT/AB/VAC is a mystery to me.		.305		.488		.461
The health of my fish in the future will depend on CT/AB/VAC on my farm.	.671		.772		.756	
CT/AB/VAC protects the fish on my farm from becoming diseased.	.672		.717		.567	
% Variance explained	43.4%		45.7%		45.9%	
Reliability (Cronbach's alpha)	.66	.34	.63	.52	.51	.54
Reliability excluding item 'CT/AB/VAC is a mystery to me'		.40		.55		.56

Note: BMQ=Beliefs about Medication (treatment) questionnaire; CT=Chemical treatment of water; AB=Antibiotics; VAC=Vaccination; Nec=Necessity subscale; Conc=Concerns subscale

In all instances the item 'CT/AB/VAC is a mystery to me' did not load as highly on the concerns subscale as the remaining three items. Reliability of the concerns scale was slightly improved with the removal of this item for all three measures.

Confirmatory principle components factor analysis was conducted for the VAX specifying four factors to be extracted and using Direct Oblimin rotation (as the four subscales are expected to be correlated). Results are shown in Supplementary Table S2.

The item 'I do not think vaccination programs are honest or trustworthy' did not load on the concerns over profiteering scale as expected from previous research, but rather (in the reverse direction) on general mistrust of vaccines. This may be due to the difficulties experienced in translating this item for the Vietnamese culture, and no doubt contributes to the low reliability of this two-item subscale.

Supplementary Table S2: Confirmatory factor analysis of the VAX questionnaire

	Mistrust	Future	Profiteering	Natural Immunity
I feel that farmed fish are safe after being vaccinated.	-.824			
I feel that farmed fish are protected after getting vaccinated.	-.842			
Although most vaccines in farmed fish appear to be safe, there may be problems that we do not know about at present.		.916		
Vaccines can cause unforeseen problems in farmed fish.		.886		
Vaccines make a lot of money for pharmaceutical companies, but don't do much for regular fish farmers.			.962	
I do not think vaccination programs are honest or trustworthy.	.605			
Vaccines only work for a short time, while natural resistance to disease in farmed fish lasts longer.				.846
Building up natural resistance to disease in farmed fish is safer than vaccination.				.849
% Variance explained	74.6%			
Reliability (Cronbach's alpha)	.63	.77	.34	.62

Attitudes by types of establishment and region

The results of oneway ANOVAs by type of farm (nursery/growout), type of farm ownership (company/contract/independent) and region are shown Table S3.

There were some differences on the BIPQ scales by farm type, with those from nursery only farms tending to have more negative views of fish disease but also a higher belief in the effectiveness of treatment. Those from combined establishments tended to have more positive views of vaccination (including higher necessity, less mistrust, less concern about profiteering) than nursery and/or growout; however they had higher concerns about future use of vaccines than nursery. The numbers in the combined category were small, and so findings may not be generalisable.

There were a few significant differences by type of farm ownership: those from company-owned farms felt they were more able to control fish disease than those from independent establishments; but they also reported worse emotional effects of fish disease. Those from company-owned farms also saw less necessity for the chemical treatment of water than independent and contract establishments and were more likely to have concerns outweighing necessity with regard to chemical treatment than contract farms. They also had more mistrust of the future use of vaccines than contract farms. Again the number of company-owned and contract establishments were quite low, meaning these findings may not be generalisable.

Table S3: Attitudes by type of establishment and province (mean (s.d.), n=400)

	Comb-ined (C)	Growout (G)	Nursery (N)	F (2,397), p, post-hoc Sheffe	Company (C)	Contract (T)	Indepen-dent (I)	F (2,397), p, post-hoc Sheffe	An Giang (A)	Can Tho (C)	Dong Thap (D)	F (2,397), p, post-hoc Sheffe
n	*13*	*191*	*196*		*16*	*39*	*345*		*163*	*92*	*145*	
BIPQ 1	4.6 (1.9)	5.1 (2.1)	6.0 (1.7)	11.7, <.001, N>C	5.6 (1.9)	4.8 (2.2)	5.6 (1.9)	3.3, .038	5.9 (1.6)	4.1 (2.0)	6.0 (2.0)	35.2, <.001, C<A,D
BIPQ 2	2.1 (0.9)	2.2 (0.7)	2.4 (0.7)	6.0, .003	2.0 (0.5)	2.1 (0.7)	2.3 (0.7)	2.9, .055	2.4 (0.8)	2.0 (0.7)	2.3 (0.7)	8.0, <.001, C<A,D
BIPQ 3	5.8 (1.5)	6.3 (1.8)	6.2 (1.5)	0.9, .391	7.3 (1.5)	6.8 (1.6)	6.1 (1.6)	6.5, .002, C>I	5.5 (1.3)	6.8 (1.5)	6.8 (1.7)	38.0, <.001, A<C,D
BIPQ 4	5.9 (1.6)	6.8 (1.5)	6.8 (1.4)	2.9, .056, N,G>C	7.1 (1.0)	6.8 (1.5)	6.8 (1.4)	0.5, .623	6.2 (1.2)	7.2 (1.3)	7.2 (1.5)	27.8, <.001, A<C,D
BIPQ 5	5.0 (1.6)	5.2 (1.8)	6.1 (1.6)	14.4, <.001, N>C	5.5 (1.8)	4.8 (1.7)	5.7 (1.8)	5.3, .005	6.0 (1.4)	4.4 (1.8)	6.0 (1.8)	35.3, <.001, C<A,D
BIPQ 6	5.1 (2.1)	5.4 (2.1)	6.2 (1.9)	9.5, p<.001	5.6 (1.4)	5.1 (2.3)	5.8 (2.0)	2.5, .082	6.0 (1.7)	4.4 (2.3)	6.3 (1.9)	32.2, <.001, C<A,D
BIPQ 7	6.8 (2.0)	7.3 (1.2)	6.9 (1.5)	4.3, .014	7.4 (1.2)	7.4 (1.5)	7.1 (1.4)	1.7, .187	6.8 (1.4)	7.4 (1.1)	7.3 (1.6)	8.1, <.001, A<C,D
BIPQ 8	4.2 (2.1)	4.7 (2.2)	5.8 (2.0)	14.4, <.001, N>C	6.0 (1.9)	4.4 (2.2)	5.3 (2.2)	4.0, .019, C>T	5.3 (1.7)	3.7 (2.1)	6.2 (2.3)	44.1, <.001, C<A<D
BMQ CT N	3.2 (0.7)	3.3 (0.5)	3.2 (0.5)	3.7, .026	2.9 (0.6)	3.5 (0.5)	3.2 (0.5)	7.1, <.001, C<I,T	3.2 (0.4)	3.6 (0.4)	3.0 (0.5)	40.0, <.001, D<A<C
BMQ CT C	3.3 (0.5)	3.1 (0.4)	3.3 (0.4)	3.7, .026	3.1 (0.4)	3.1 (0.4)	3.2 (0.4)	2.0, .141	3.2 (0.4)	3.1 (0.5)	3.3 (0.5)	6.9, .001, D>C
BMQ CT N-C	-0.2 (0.9)	0.2 (0.7)	-0.1 (0.7)	7.2, <.001	-0.2 (0.7)	0.4 (0.7)	0.0 (0.7)	6.1, .003 C<T	0.0 (0.6)	0.5 (0.6)	-0.3 (0.7)	43.8, <.001, D<A<C
BMQ AB N	2.8 (0.5)	2.9 (0.5)	2.9 (0.5)	0.9, .415	2.6 (0.3)	2.8 (0.6)	2.9 (0.5)	3.2, .043	2.8 (0.5)	3.1 (0.5)	2.9 (0.5)	8.1, <.001 D,A<C
BMQ AB C	3.5 (0.4)	3.3 (0.5)	3.4 (0.6)	3.3, .037	3.2 (0.7)	3.3 (0.5)	3.4 (0.5)	0.7, .520	3.4 (0.6)	3.2 (0.5)	3.4 (0.5)	4.4, .012, C<D
BMQ AB N-C	-0.7 (0.7)	-0.4 (0.8)	-0.5 (0.8)	1.0, .388	-0.6 (0.6)	-0.5 (0.8)	-0.4 (0.8)	0.4, .682	-0.5 (0.8)	-0.1 (0.8)	-0.6 (0.7)	10.4, <.001, C>A,D
BMQ VAC N	3.8 (0.3)	3.5 (0.4)	3.4 (0.4)	8.4, <.001, C>N,G	3.4 (0.4)	3.5 (0.4)	3.4 (0.4)	1.1, .335	3.5 (0.4)	3.6 (0.4)	3.3 (0.4)	11.4, <.001.D<A,C
BMQ VAC C	2.9 (0.5)	3.1 (0.4)	3.1 (0.4)	1.2, .306	3.1 (0.4)	3.0 (0.4)	3.1 (0.4)	0.7, .483	3.1 (0.4)	3.1 (0.4)	3.1 (0.4)	0.5, .636
BMQ VAC N-C	0.8 (0.7)	0.4 (0.7)	0.3 (0.7)	4.9, .008, C>N,G	0.3 (0.7)	0.5 (0.7)	0.4 (0.7)	1.3, .274	0.4 (0.7)	0.5 (0.7)	0.2 (0.6)	5.9, .023, D<C
VAX 1	2.0 (0.4)	2.2 (0.5)	2.3 (0.6)	3.8, .023, C<N	2.3 (0.5)	2.2 (0.5)	2.3 (0.5)	0.2, .807	2.2 (0.6)	2.2 (0.4)	2.3 (0.5)	2.1, .122
VAX 2	3.4 (0.6)	3.1 (0.6)	3.0 (0.6)	3.1, .048, N<C	3.3 (0.5)	2.8 (0.5)	3.1 (0.6)	4.4, .013, C<T	2.9 (0.6)	3.2 (0.6)	3.2 (0.6)	7.7, <.001 A<C,D
VAX 3	3.4 (0.3)	2.8 (0.6)	2.7 (0.5)	2.3, .099, C<N	2.7 (0.6)	2.6 (0.7)	2.8 (0.5)	2.1, .122	2.8 (0.6)	2.7 (0.5)	2.7 (0.5)	1.2, .305
VAX 4	2.9 (0.5)	2.9 (0.6)	2.9 (0.5)	0.0, .976	2.8 (0.7)	2.8 (0.6)	3.0 (0.5)	2.5, .087	3.0 (0.5)	2.9 (0.6)	2.9 (0.6)	1.1, .348

BIPQ: 1=How much does fish disease on your farm affect your life?, 2=How long do you think fish disease on your farm will last in the first production cycle (in weeks)?, 3=Do you think you are able to control fish disease on your farm?, 4=How much do you think treatment can help fish disease on your farm?, 5=How much do you experience problems from fish disease on your farm?, 6=How worried are you about fish disease on your farm?, 7=Do you think you have a good understanding of fish disease on your farm?, 8=How much does fish disease on your farm affect you emotionally?; BMQ CT=Chemical Treatment, N=Necessity, C=Concerns, N-C=Necessity minus concerns, BMQ AB= Antibiotics, BMQ VAC=Vaccination; VAX: 1=mistrust of vaccines, 2=worries over future use, 3=concerns over profiteering, 4= preference for natural immunity

There were notable differences on the attitudes measures by province (see Table S3). On the BIPQ those from Cần Thơ had less negative views of fish disease than An Giang and Đồng Tháp on all items regarding concern about disease (affects life, how long will continue, how many problems, how worried and emotional effect). Those from An Giang province had less positive views than Cần Thơ and Đồng Tháp with regard to control over disease, knowledge of disease and effectiveness of treating disease.

Regarding chemical treatment of water, those from Đồng Tháp reported less necessity than An Giang, who also reported lower necessity than those from Cần Thơ . Those from Đồng Tháp also reported more concern than those from Cần Thơ . Necessity minus concerns was significantly worse (i.e. concerns outweighed necessity) for Đồng Tháp versus An Giang, who also had lower scores than those from Cần Thơ (where necessity outweighed concerns). Similar results were found for antibiotic use, with those from Cần Thơ seeing a greater necessity (than An Giang and Đồng Tháp) and having fewer concerns (than Đồng Tháp). All regions reported that concerns re: antibiotic use outweighed necessity, but this was greatest for Đồng Tháp versus the other two regions.

Regarding vaccines, those from Đồng Tháp saw a lower necessity than the other two regions. All three regions saw necessity for vaccines outweighing concerns, but this was greatest for Cần Thơ (and significantly higher than Đồng Tháp). On the VAX scale, the only difference was that those from An Giang reported less worry about future use than the other two regions.

It is possible that the views of those from Cần Thơ with regard to necessity of treatments (i.e. higher necessity versus concerns) are related to them reporting fewer problems with fish disease (as evidenced by the BIPQ responses).

Table S4: Correlations between attitudes measures (Pearsons r, n=400)

	BIPQ								BMQ CT			BMQ AB			BMQ VAC			VAX		
	1	2	3	4	5	6	7	8	Nec	Con	N-C	Nec	Con	N-C	Nec	Con	N-C	1	2	3
BIPQ 1																				
BIPQ 2	.12*																			
BIPQ 3	-.16*	-.08																		
BIPQ 4	.05	-.06	.47**																	
BIPQ 5	.57**	.05	-.11#	.07																
BIPQ 6	.60**	.07	-.10#	.07	.63**															
BIPQ 7	-.01	-.10	.32**	.31**	-.03	.12#														
BIPQ 8	.58**	.07	-.01	.08	.49**	.70**	.08													
BMQ CT N	-.16*	-.03	-.05	-.09	-.23**	-.20**	-.02	-.27**												
BMQ CT C	.04	.07	-.07	-.02	.14*	.05	-.09	.03	-.02											
BMQ CT N-C	-.15*	-.07	.01	-.06	-.27**	-.18**	.07	-.22**	.77**	-.65**										
BMQ AB N	.08	-.03	-.01	.02	.02	-.05	-.03	-.07	.32**	.09	.18**									
BMQ AB C	.02	-.05	-.07	-.06	.16*	.06	-.05	.02	.06	.46**	-.25**	-.14*								
BMQ AB N-C	.03	.01	.05	.06	-.09	-.08	.01	-.06	.17*	-.26**	.29**	.74**	-.76**							
BMQ VAC N	-10#	-.04	-.00	-.07	-.18**	-.11#	.04	-.16*	.18*	-.11#	.21**	.01	.01	-.00						
BMQ VAC C	-.02	.10	-.03	.02	-.01	-.09	-.14*	-.08	.03	.23**	-.12#	.01	.12#	-.09	-.37**					
BMQ VAC N-C	-.05	-.08	.01	-.06	-.10#	-.01	.11#	-.05	.09	-.21**	.20**	.01	-.06	.05	.83**	-.82**				
VAX 1	.08	.01	-.05	.03	.10#	.06	-.07	.02	-.05	.12#	-.11#	-.01	.01	-.01	-.61**	.35**	-.58**			
VAX 2	-.04	-.05	.00	.04	-.04	-.00	-.05	-.04	.05	.10#	-.03	.03	.01	.01	-.25**	.25**	-.30**	.28**		
VAX 3	-.02	.04	-.07	.06	.05	.05	-.08	.03	-.02	.12#	-.09	.01	-.04	.03	-.38**	.38**	-.46**	.36*	.27**	
VAX 4	-.08	.06	-.01	.08	-.07	-.04	-.10	-.07	-.05	.07	-.08	-.00	-.05	.03	-.13*	.22**	-.21**	.14*	.18**	.25**

#p<.05, *p<.01, **p<.001; BIPQ: 1=How much does fish disease on your farm affect your life?, 2=How long do you think fish disease on your farm will last in the first production cycle (in weeks)?, 3=Do you think you are able to control fish disease on your farm?, 4=How much do you think treatment can help fish disease on your farm?, 5=How much do you experience problems from fish disease on your farm?, 6=How worried are you about fish disease on your farm?, 7=Do you think you have a good understanding of fish disease on your farm?, 8=How much does fish disease on your farm affect you emotionally?; BMQ CT=Chemical Treatment, N=Necessity, C=Concerns, N-C=Necessity minus concerns, BMQ AB= Antibiotics, BMQ VAC=Vaccination; VAX: 1=mistrust of vaccines, 2=worries over future use, 3=concerns over profiteering, 4= preference for natural immunity

File S1: Study questionnaire

VAAC Survey

PART 1: Fish disease and its treatment questionnaire

Presentation of Attitude Questionnaire

"This research project aims to help us understand the views of fish farmers in Vietnam regarding different ways of treating fish disease.

We would like to ask you some questions about fish disease and its treatment on your farm.

We are interested in your personal views. There are no right or wrong answers to the questions and your answers will be confidential, will not be linked to your identity and will not be seen by anyone outside of the research team. Your answers will be very useful in helping us to understand how fish farmers feel about fish disease and its treatment".

Subject ID: V_____

Participant Details

Survey Date:

Full Name:

Initials:

Farm:

Farm-role? (farm manager, farm owner, farm worker, other)

Tel No.:

Email:

Nursery only/Grow-out only/Combined?

Independent/Contract/Company?

Province (An Giang/Đồng Tháp/Cần Thơ)?

Questionnaire 1 (Adapted Brief Illness Perceptions Questionnaire - BIPQ)

We would like to ask some questions about your experience of fish disease on your farm. There are no right or wrong answers; we are just interested in your personal views, whether you have had fish disease on your farm or have not had fish disease. Please indicate the number that best corresponds to your personal views [*if no disease then use options in brackets below*]

Item	Question
**BIPQ1**	**How much does fish disease on your farm affect your life?**<br>0 1 2 3 4 5 6 7 8 9 10<br>no affect at all — severely affects my life
**BIPQ2**	**How long do you think fish disease on your farm will last in the first production cycle (in weeks)?** [*if no disease then circle* 0]<br>0 1 2 3 4 5 6 7 8 9 10<br>0 weeks — 10 or more weeks
**BIPQ3**	**Do you think you are able to control fish disease on your farm?**<br>0 1 2 3 4 5 6 7 8 9 10<br>absolutely cannot control — definitely can control
**BIPQ4**	**How much do you think treatment can help fish disease on your farm?** [*if no disease then:* **How much do you think treatment will help fish disease on your farm if you had disease in the future?]**<br>0 1 2 3 4 5 6 7 8 9 10<br>not at all helpful — extremely helpful
**BIPQ5**	**How much do you experience problems from fish disease on your farm?**<br>0 1 2 3 4 5 6 7 8 9 10<br>no problems at all — many severe problems
**BIPQ6**	**How worried are you about fish disease on your farm?**<br>0 1 2 3 4 5 6 7 8 9 10<br>not at all worried — extremely worried
**BIPQ7**	**Do you think you have a good understanding of fish disease on your farm?**<br>0 1 2 3 4 5 6 7 8 9 10<br>don't understand at all — understand very clearly

**BIPQ8**	**How much does fish disease on your farm affect you emotionally? (e.g. does it make you angry, scared, up-set or depressed?**
	0 1 2 3 4 5 6 7 8 9 10
	not at all affected emotionally — extremely affected emotionally

Questionnaire 2 (Adapted Beliefs about Medicines Questionnaire - BMQ)

We would like to ask your personal views about **the use of chemical treatment of the water** on your fish farm. These are statements other people have made about different treatments.

First, have you used chemical treatment on your farm [*circle their answer*]?

[CHEMTREAT] Yes No

Please indicate the extent to which you agree or disagree with each statement. There are no right or wrong answers. We are interested in your personal views. *[If they feel they can't answer any question (for example have not used chemical treatment) then circle 'not sure' for that item].*

**ITEM**	**MY BELIEFS ABOUT CHEMICAL TREATMENT OF WATER**	STRONGLY DISAGREE	DISAGREE	NOT SURE	AGREE	STRONGLY AGREE
**BMQCT1**	The health of my fish depends on the use of chemical treatment of the water on my farm.	1	2	3	4	5
**BMQCT2**	Having to use chemical treatment of the water on my fish farm worries me.	1	2	3	4	5
**BMQCT3**	Without chemical treatment of the water the fish on my farm would be very sick.	1	2	3	4	5
**BMQCT4**	I sometimes worry about the long-term effects of using chemical treatment of the water on my farm.	1	2	3	4	5
**BMQCT5**	Chemical treatment of water is a mystery to me.	1	2	3	4	5
**BMQCT6**	The health of my fish in the future will depend on chemical treatment of the water on my farm.	1	2	3	4	5
**BMQCT7**	Chemical treatment of the water protects the fish on my farm from becoming diseased.	1	2	3	4	5
**BMQCT8**	I sometimes worry about becoming too dependent on using chemical treatment of the water on my fish farm.	1	2	3	4	5

Next we would like to ask the same questions regarding your personal views about **the use of antibiotics** in fish on your farm.

First, have you used antibiotics on your farm [*circle their answer*]?

[ANTIBIOTICS] Yes No

Please indicate the extent to which you agree or disagree with each statement. There are no right or wrong answers. We are interested in your personal views. *[If they have feel they can't answer any question then circle 'not sure' for that item].*

ITEM	MY BELIEFS ABOUT USING ANTIBIOTICS	STRONGLY DISAGREE	DISAGREE	NOT SURE	AGREE	STRONGLY AGREE
**BMQAB1**	The health of fish on my farm depends on antibiotics.	1	2	3	4	5
**BMQAB2**	Having to use antibiotics in fish on my farm worries me.	1	2	3	4	5
**BMQAB3**	Without antibiotics my fish would be very sick.	1	2	3	4	5
**BMQAB4**	I sometimes worry about the long-term effects of using antibiotics in the fish on my farm.	1	2	3	4	5
**BMQAB5**	Antibiotics are a mystery to me.	1	2	3	4	5
**BMQAB6**	The health of my fish in the future will depend on antibiotics.	1	2	3	4	5
**BMQAB7**	Antibiotics protect the fish on my farm from becoming diseased.	1	2	3	4	5
**BMQAB8**	I sometimes worry about becoming too dependent on using antibiotics in the fish on my farm.	1	2	3	4	5

We would now like to ask some questions about **your understanding of vaccination**. Please indicate the answer which best represents your understanding [*circle their answers]*

**ITEM**	**Question**
	Do you understand what is meant by vaccinating people?

**[VACCINES1]**	Yes	No	Not sure	

Do you know that vaccines can be used in fish?

**[VACCINES2]**	Yes	No	Not sure	

How do you think vaccines work?

**[VACCINES3]**	By treating disease	By preventing disease	By both treating and preventing disease	Not sure

Have you used vaccines on your farm?

**[VACCINES4]**	Yes	No

Would use vaccines on your farm if they were available?

**[VACCINES5]**	Yes	No	Not sure

Now we would like to ask some familiar questions, but this time regarding your personal views about **the use of vaccines in fish on your farm.**

Please indicate the number which represents the extent to which you agree or disagree with each statement. There are no right or wrong answers. We are interested in your personal views. *[If they have feel they can't answer any question then circle 'not sure' for that item].*

ITEM	MY BELIEFS ABOUT USING VACCINES ON MY FARM	STRONGLY DISAGREE	DISAGREE	NOT SURE	AGREE	STRONGLY AGREE
**BMQVAC1**	The health of my fish depends on vaccination.	1	2	3	4	5
**BMQVAC2**	Having to use vaccination in fish on my farm worries me.	1	2	3	4	5
**BMQVAC3**	Without vaccination my fish would be very sick	1	2	3	4	5
**BMQVAC4**	I sometimes worry about the long-term effects of using vaccination in the fish on my farm.	1	2	3	4	5
**BMQVAC5**	Vaccination is a mystery to me.	1	2	3	4	5
**BMQVAC6**	The health of fish on my farm in the future will depend on vaccination.	1	2	3	4	5
**BMQVAC7**	Vaccination protects the fish on my farm from becoming diseased.	1	2	3	4	5
**BMQVAC8**	I sometimes worry about becoming too dependent on using vaccination in the fish on my farm.	1	2	3	4	5

Questionnaire 3 (Adapted Vaccination Attitudes Examination (VAX) Scale)

Finally, we have some questions which are designed to help us better understand people's beliefs about **vaccination in farmed fish** in general, not only on your fish farm. Please indicate the number that most accurately reflects your feelings or beliefs. There are no right or wrong answers [*if they feel they don't know enough to answer any question then circle 'not sure' for that item*].

ITEM	MY BELIEFS ABOUT USING VACCINES IN FARMED FISH IN GENERAL	STRONGLY DIS-AGREE	DISAGREE	NOT SURE	AGREE	STRONGLY AGREE
**VAX1**	I feel that farmed fish are safe after being vaccinated.	1	2	3	4	5
**VAX2**	I feel that farmed fish are protected after getting vaccinated.	1	2	3	4	5
**VAX3**	Although most vaccines in farmed fish appear to be safe, there may be problems that we do not know about at present.	1	2	3	4	5
**VAX4**	Vaccines can cause unforeseen problems in farmed fish.	1	2	3	4	5
**VAX5**	Vaccines make a lot of money for pharmaceutical companies, but don't do much for regular fish farm-ers.	1	2	3	4	5
**VAX6**	I do not think vaccination programs are honest or trustworthy.	1	2	3	4	5
**VAX7**	Vaccines only work for a short time, while natural resistance to disease in farmed fish lasts longer.	1	2	3	4	5
**VAX8**	Building up natural resistance to disease in farmed fish is safer than vaccination.	1	2	3	4	5

Thank you very much.

Would you be interested in taking part in a short interview about fish disease and its treatment at a later date?

[INTERVIEW] Yes No

End of Part 1

PART 2: Behavioural Economics Survey

<table>
<tr><td>Introduction</td><td>Scientists have been working for several years to develop vaccines that can prevent the two most common diseases in farmed catfish.

The first is sometimes called “White Spot” and its scientific name is bacillary necrosis.

(data collector shows photo of White Spot)

The second is sometimes called “Red Spot” or “haemorrhage disease” and its scientific name is motile aeromonas septicaemia (MAS).

(data collector shows photo of Red Spot)

This survey is to learn whether you would buy vaccines to prevent these diseases in catfish if vaccines were available on the market.

You would give the vaccine to the fish to prevent the fish from getting these diseases at any point in the production cycle. Any fish weighing between 0.5 and 5 grams could receive the vaccine. The vaccine would not be effective in treating fish who already have the disease or that have other diseases.</td></tr>
<tr><td>Q1.</td><td>Who makes the decision about how to deal with an outbreak of disease in your fish?

☐ Myself alone
☐ Some other individual
☐ A committee of other individuals
☐ A committee that includes myself</td></tr>
<tr><td>Explaining attributes</td><td>You will shortly be asked whether you would choose to buy a vaccine to treat fish that you farm.

You will be asked whether you would buy various different vaccines. The vaccines differ in the following characteristics:
- Whether the vaccines protect against White Spot, Red Spot or both diseases
- How the vaccines are delivered to the fish: by bath or by injection
- Price you would pay to buy the vaccine
- Effectiveness of the vaccine

Let’s start by discussing effectiveness, because it is more complicated than the other characteristics.</td></tr>
</table>

Explaining attrib-utes	Effectiveness is the term for how well a vaccine protects against getting a spe-cific disease.<br>As an example, consider a vaccine which is 70% effective in preventing a dis-ease over the production cycle of a fish. I want to explain exactly what I mean when I say the vaccine would be 70% effective.
Training	Please take a look at this diagram.<br><br>(*Data collector hands* Card A *to the respondent*).<br><br>There are 100 fish in this diagram. If a vaccine is 70% effective, that means that we would expect 70 out of 100 vaccinated fish to be protected (i.e., the vaccine works for them). The blue fish in the diagram represent these fish. They CANNOT get the disease or pass it on to other fish at any point over the production cycle.<br><br>The rest of the fish (the 30 red ones) will not be protected against the disease even though they have gotten the vaccine, because the vaccine did not work for them. They will still be at risk of getting the disease just like they were be-fore they got the vaccine. Of course, we don't know which fish would actually get the disease.
Q.B.1.	I now have to ask you three questions to make sure you understand effective-ness.<br><br>(*Make sure the respondent still has Card A*)<br><br>In the diagram, what color are the fish that the vaccine has successfully pro-tected against disease?<br>☐ Blue<br>☐ Red
Q.B.2.	How many fish out of 100 have received the vaccine but can still get the dis-ease?<br><br>Respondent's answer ______
Q.B.3.	If a fish gets infected with White Spot, can the vaccine against White Spot be used to cure them?<br>[*interviewer: tick box for the respondents' answer below*]<br>☐ Yes<br>☐ No
Introducing alter-natives	Card A, which you are holding, shows a vaccine with 70% effectiveness against White Spot.<br><br>Here is card B, which depicts a different vaccine.

Q.C.1	Which card shows the vaccine that is more effective at protecting against White Spot, A or B?<br>[*interviewer: tick box for the respondents' answer below*]<br>☐ A<br>☐ B
Q.C.2	As well as effectiveness, vaccines can differ because some protect against White Spot only, some protect against Red Spot only and some protect against both diseases.<br><br>Make sure to pay attention to which diseases the vaccine protects against.<br><br>The vaccine depicted on card B protects against which diseases?<br><br>☐ Red Spot only<br>☐ White Spot only<br>☐ Both
Delivery method	Another feature that might matter to you is how the vaccine is delivered to the fish.<br><br>A vaccine can be delivered by bath or by injection.<br><br>If the vaccine is delivered by bath then each fish needs to be dunked in a bath that contains the vaccine. You only need to dunk each fish once.<br><br>If the vaccine is delivered by injection then each fish needs to receive an injection of the vaccine under its ribs. You only need to inject each fish once.
Price	Vaccines can also differ in price.<br><br>The price listed on the card is the price of buying the vaccine. It does not cover the costs of delivering the vaccine to the fish – that is something that your farm will have to pay and you should consider those costs when making your choice.<br><br>Card A depicts a vaccine that is 70% effective in protecting against White Spot only, that is delivered by injection and that costs 300,000 dong per thousand fish.<br><br>Card B depicts a vaccine that is 80% effective in protecting against White Spot only, that is delivered by bath and that costs 500,000 dong per thousand fish.

Q.D.1.	Now, let's suppose that these vaccines were available for you to purchase. Which one would you purchase?<br><br>☐ A<br>☐ B<br>☐ Neither *(if they chose neither ask next question; otherwise read the final "*choice experiment*" text below)*
Qualitative fol-low up if chose neither	Why did you choose neither? (*data collector, allow respondent explain; don't write, just listen*)
Q.D.2	Thanks for that explanation. Which sentence below best explains why you chose "neither" instead of vaccine A or vaccine B?<br><br>☐ Disease is unimportant<br>☐ Disease is important but it is too expensive to treat with these vaccines<br>☐ Disease is important but it is too inconvenient to treat with these vaccines<br>☐ I already have a more convenient and cheaper way of treating disease
Choice experi-ment	We now have a series of choices for you to make, just like the choice between Vaccine A and Vaccine B that you made a moment ago.<br><br>Please take the following booklet – each page of it offers you a choice of buy-ing or not buying some Vaccine A.<br><br>Your choices to these questions will help us to identify your preferences and ultimately help us to deliver a vaccine that suits the preferences of you and other fish farmers.<br><br>When you have completed the 8 choices return your booklet to us.
